# A 5-year-old with new-onset diabetes presenting with ketoacidosis, acute pancreatitis, and renal failure

**DOI:** 10.1177/2050313X221130582

**Published:** 2022-10-15

**Authors:** Seth M Alexander, Vivek Shenoy, Margaret Kihlstrom, Amy Levenson

**Affiliations:** 1 Office of Medical Education, University of North Carolina School of Medicine, Chapel Hill, NC, USA; 2 Division of Pediatric Gastroenterology, Department of Pediatrics, University of North Carolina School of Medicine, Chapel Hill, NC, USA; 3 Division of Pediatric Critical Care, Department of Pediatrics, University of North Carolina School of Medicine, Chapel Hill, NC, USA; 4 Division of Pediatric Endocrinology and Diabetes, Department of Pediatrics, University of North Carolina School of Medicine, Chapel Hill, NC, USA

**Keywords:** Diabetes, endocrinology, gastroenterology, critical care, emergency medicine, pancreatitis, diabetic ketoacidosis, shock, renal failure

## Abstract

A 5-year-old girl presented to the emergency room with altered mental status secondary to severe diabetic ketoacidosis due to new-onset GAD65 antibody positive, type 1 diabetes mellitus. On hospital day 0, she developed anuria, shock, and hypertriglyceridemia-associated acute pancreatitis. Following intravenous insulin therapy, the patient’s ketoacidosis improved. Her other complications persisted for several days and improved only with significant fluid resuscitation and supportive interventions, including intubation, thoracostomy, and vasopressors. This case underscores the importance of recognizing the early warning signs of diabetic ketoacidosis and reviews how to appropriately manage its associated life-threatening complications.

## Introduction

Typical symptoms of new-onset type 1 diabetes mellitus include weight loss, polyuria, and polydipsia.^1^ In pediatric patients who are not gaining appropriate weight, and especially in those that are losing weight, evaluation for hyperglycemia is crucial. Of newly diagnosed type 1 diabetes cases in the United States, 13%–80% will present initially in diabetic ketoacidosis (DKA).^2,3^ DKA has an estimated mortality rate in the United States of 1%–4% with similar rates seen in other nations with developed healthcare infrastructures.^2^ Severe complications include cerebral edema (21%–24% mortality rate), stroke, and hypovolemic shock.^2–6^ Another complication relevant to the case presented is hypertriglyceridemia-induced acute pancreatitis. While thoroughly reported in adults and type 2 diabetes mellitus, hypertriglyceridemia-induced acute pancreatitis has been described less than 20 times in the pediatric type 1 diabetes mellitus literature.^3,7,8^ Although pancreatic enzyme elevation is common in DKA, diagnostic criteria for pancreatitis are met in 2% or less of cases.^9^

## Case

A 5-year-old, previously healthy female presented to an outside emergency department with non-bilious, non-bloody vomiting and loss of consciousness following approximately 3 months of poor weight gain, polyuria, and polydipsia. Her parents reported having been concerned that she was not gaining weight for several months prior to presentation. She had no sick contacts, fevers, chills, or other significant symptoms.

Within several hours from the onset of nausea and vomiting, her parents took her to the emergency room to be evaluated. En route to the community hospital, the patient lost consciousness. Labs at the emergency room were significant for a pH of 6.88, glucose of 610 mg/dL (reference: 70–179 mg/dL), bicarbonate of <1.9 mmol/L (reference: 22.0–33.0 mmol/L), and urine ketones of 80 mg/dL (reference: <0.1 mg/dL). Taken together, these findings were consistent with DKA. Given her altered mental status, she required intubation to protect her airway. She was given an isotonic fluid bolus of approximately 10 mL/kg, started on an insulin infusion, and transferred to a regional medical center (RMC).

On admission to the RMC, venous blood gas was significant for a pH of 6.78, pCO_2_ of 33.9 mm Hg (reference: 40–60 mm Hg), and bicarbonate of 5 mmol/L (reference: 22–27 mmol/L). Her serum sodium was 128 mmol/L (reference: 135–145 mmol/L), potassium 3.8 mmol/L (3.4–4.7 mmol/L), chloride 110 mmol/L (98–107 mmol/L), blood urea nitrogen 21 mg/dL (reference: 5–17 mg/dL), creatinine < 0.40 mg/dL (reference: 0.45–1.0 mg/dL), and glucose > 700 mg/dL (70–179 mg/dL). She was extubated on hospital day 0 given her improved mental status. The laboratory at the RMC also reported difficulty processing her labs due to profound hypertriglyceridemia (> 1100 mg/dL, reference: 32–105 mg/dL) (Table 1).

**Table 1.** Summary of laboratory values throughout the patient’s care. Records prior to admission to the RMC are limited. Due to the profound lipemia, samples taken at the RMC and Quaternary center were ultracentrifuged. Reported results are directly taken from the laboratory assays and not corrected for hyperglycemia or hypertriglyceridemia.

	Reference range (quaternary lab)	At presentation to local hospital (hospital day 0)	On admission to the RMC (hospital day 0)	Transfer to the quaternary center (hospital day 1)
Blood gas, arterial				
pH	7.35–7.45	6.88	6.78	6.98
pCO_2_	35–45 mm Hg		33.9	31.8
pO_2_	80–110 mm Hg		53	114
Bicarbonate	22–27 mmol/L	<1.9	5	8
FiO2	%			40
MAP	mm Hg			15.1
Metabolic panel				
Sodium	135–145 mmol/L		128	140
Potassium	3.4–4.7 mmol/L		3.8	4.7
Chloride	98–107 mmol/L		110	123
BUN	5–17 mg/dL		21	30
Creatinine	0.45–1.0 mg/dL		<0.40	1.46
Glucose	70–179 mg/dL	610	>700	304
Urine ketones	<0.1 mg/dL (negative)	80		Negative
Beta-hydroxybutyrate	0.2–2.8 mg/dL		1.5	1.5
Hemoglobin A1c	<5.7%		12.8	
Islet cell antibodies	Negative		Negative	
Endomysial antibodies	Negative		Negative	
GAD-65 antibodies	0–5 U/mL		7588.8	
Triglycerides	32–105 mg/dL		>1100	2841
Lipase, serum	10–150 U/L			2599

RMC: regional medical center; BUN = blood urea nitrogen..

Later on hospital day 0, the patient’s abdomen became tense. An ultrasound showed heterogeneous pancreatic parenchyma with surrounding peripancreatic fluid without evidence of gallstones or choledocholithiasis. Subsequent labs showed serum lipase of 2599 U/L (reference: 10–150 U/L), consistent with acute pancreatitis. The patient then had an episode of emesis accompanied by significant oxygen desaturation; she was reintubated for acute hypoxemic respiratory failure. Following reintubation, she became profoundly hypotensive, requiring an epinephrine infusion to maintain adequate perfusion. The patient’s urine output, monitored via urinary catheter, decreased despite fluid resuscitation and furosemide therapy. Given her declining clinical status, the patient was referred to our quaternary center on hospital day 1 for possible dialysis given her anuria and extracorporeal membrane oxygenation (ECMO) for persistent hemodynamic instability.

**Figure 1.**
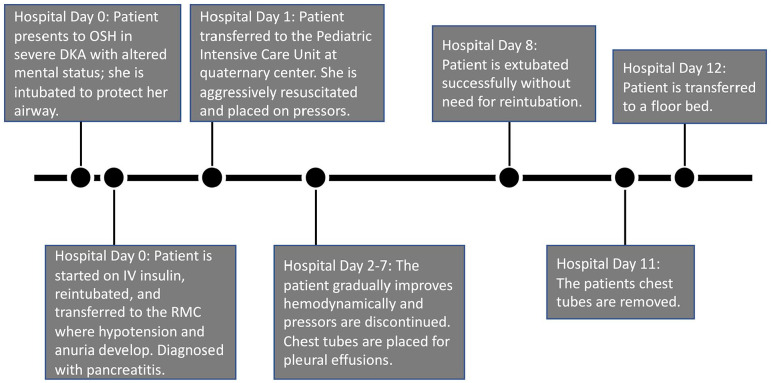
Timeline summary of the patient’s hospital course from presentation to the outside, community hospital until transfer to the floor at the quaternary medical center.

The patient was admitted to our Pediatric Intensive Care Unit (PICU) and evaluated by a multidisciplinary team. Her presentation was most concerning for shock secondary to hypovolemia and comorbid pancreatitis with mild acute respiratory distress syndrome (ARDS, oxygenation saturation index 6.1).^10^ She required ongoing fluid resuscitation and vasopressor therapy with norepinephrine, epinephrine, and dobutamine infusions. She had improvement in urine output and blood pressure with fluid resuscitation; however, she developed bilateral pleural effusions requiring thoracostomy in the setting of her pancreatitis and capillary leak, among other possible etiologies. Insulin infusion normalized her serum triglyceride levels by hospital day 3, and her abdominal pain resolved. She had gradual improvement in her vital signs and pleural effusions. She was extubated on hospital day 8 and her thoracostomy tubes were removed on day 11 (Figure 1).

Although the blood glucose had normalized and anion gap had closed prior to transfer to the quaternary medical center, she was continued on intravenous insulin infusion until hospital day 5 to prevent recurrent hyperglycemia and acidosis. She was then transitioned to a subcutaneous, multidose insulin regimen. Once she was extubated and eating, her insulin regimen was tailored to her glycemic needs. Further laboratory studies showed a hemoglobin A1c of 11.8% (reference: 4.8%–5.6%), C-peptide of 0.6 ng/mL (reference: 1.1–4.4 ng/mL), and positive GAD65 antibodies. The patient had fully recovered by her first outpatient follow-up with pediatric endocrinology. She subsequently transferred care to a local medical institution and outpatient laboratory follow-up was unavailable. Additional genetic and familial testing for other possible etiologies of the hypertriglyceridemia was deferred due to a negative family history and normalization of triglycerides prior to discharge.

## Discussion

This case emphasizes the warning signs of type 1 diabetes, classically polydipsia, polyphagia, and polyuria, as well as the steps of DKA treatment and its complications. DKA is a severe, life-threatening condition caused by severe insulin deficiency from β -cell failure, insulin dysfunction, or another stressor. These causes lead to an increase in counterregulatory hormones which promote lipolysis, proteolysis, glycogenolysis, and decreased glucose utilization. The inability of the body to utilize glucose produced through these mechanisms results in hyperglycemia. Hyperglycemia induces a hyperosmolar state leading to osmotic diuresis and dehydration. Ketoacidosis develops as a consequence of lipolysis triggering the release of free fatty acids (FFAs) which are metabolized to ketoacids such as β -hydroxybutyrate and acetoacetate.^2,3^

In addition to hyperglycemia and ketoacidosis, lipoprotein metabolism is often altered in DKA. Insulin promotes lipolytic action via induction of lipoprotein lipase (LPL). In insulin deficient states, LPL activity is reduced, resulting in increased serum triglycerides.^11,12^ Hyperviscosity, from elevated lipids, may lead to poor pancreatic perfusion with subsequent ischemia and inflammatory response resulting in acute pancreatitis. Triglyceride catabolism to FFAs may also cause cytotoxic effects on pancreatic acinar cells resulting in acute pancreatitis.^7,13^ Acute pancreatitis is life-threatening as the inflammation can induce systemic inflammatory response syndrome (SIRS) leading to distributive shock if unmanaged.^14^ In the setting of comorbid DKA, it is critical to address the patient’s mixed distributive and hypovolemic shock as well as their hyperglycemic ketoacidosis.

Initial resuscitation efforts in DKA should focus on the patient’s immediate respiratory and cardiovascular needs. In this case, intubation was necessary due to her altered mental status and inability to protect her airway. Altered mental status due to cerebral edema is present in up to 0.3% of DKA cases,^2–4,6^ but carries a significant risk of mortality.^2,4^ No head imaging was performed in this patient prior to intubation; it is presumed her altered mental status was secondary to multiple factors. Once immediate interventions are implemented, other treatments can proceed, including rapid and adequate fluid resuscitation. The degree of rehydration is controversial given that resuscitation is necessary to prevent hypovolemic shock, but volume overload can increase the risk for cerebral edema and/or exacerbate complications of capillary leak in pro-inflammatory states.^5,15^ In instances of cerebral edema, hypertonic fluids may be necessary to avoid tissue injury and subsequent mortality; these therapies are not, however, without their own independent risks and remain controversial.^16,17^ In this patient, further fluid resuscitation was necessary to support the patient’s blood pressure and renal function. Furthermore, many patients with acute renal failure and comorbid DKA improve with fluid resuscitation; however, some require hemodialysis as therapy for resistant cases of acidosis, which should be considered only after ensuring adequate therapy has been given.^18–20^

The primary therapy for DKA is insulin infusion to resolve the patient’s hyperglycemia and ketoacidosis. In cases with comorbid pancreatitis secondary to hypertriglyceridemia, intravenous insulin can be used to degrade triglycerides by stimulating lipoprotein lipase and should be continued until levels are <500 mg/dL.^2,3,8,12^ Treating hypertriglyceridemia should aid in the resolution of the pancreatitis. While this patient improved without further intervention, therapeutic plasma exchange has been reported to reduce triglyceride levels in the treatment of pancreatitis in refractory cases.^21,22^ Pancreatitis has also been associated with the development of acute respiratory distress syndrome (ARDS) and pleural effusions requiring thoracostomy for acute relief and treatment of the underlying cause is necessary to prevent further respiratory impairment.^23^

## Conclusion

This case of DKA presented with a severe constellation of hypovolemic and distributive shock requiring aggressive intervention. Care of patients presenting with such severe shock should involve immediate, life-preserving interventions along with aggressive investigation to determine the underlying causes. As in this case, care should be multidisciplinary, involving pediatric critical care, endocrinology, gastroenterology, and other subspecialists to fully address the needs of the patient.
